# An Elastic Self-Adjusting Technique for Rare-Class Synthetic Oversampling Based on Cluster Distortion Minimization in Data Stream

**DOI:** 10.3390/s23042061

**Published:** 2023-02-11

**Authors:** Hayder K. Fatlawi, Attila Kiss

**Affiliations:** 1 Department of Information Systems, ELTE Eötvös Loránd University, 1117 Budapest, Hungary; 2 Center of Information Technology Research and Development, University of Kufa, Najaf 540011, Iraq; 3 Department of Informatics, J. Selye University, 94501 Komárno, Slovakia

**Keywords:** adaptive machine learning, cluster analysis, imbalance data stream, SMOTE

## Abstract

Adaptive machine learning has increasing importance due to its ability to classify a data stream and handle the changes in the data distribution. Various resources, such as wearable sensors and medical devices, can generate a data stream with an imbalanced distribution of classes. Many popular oversampling techniques have been designed for imbalanced batch data rather than a continuous stream. This work proposes a self-adjusting window to improve the adaptive classification of an imbalanced data stream based on minimizing cluster distortion. It includes two models; the first chooses only the previous data instances that preserve the coherence of the current chunk’s samples. The second model relaxes the strict filter by excluding the examples of the last chunk. Both models include generating synthetic points for oversampling rather than the actual data points. The evaluation of the proposed models using the Siena EEG dataset showed their ability to improve the performance of several adaptive classifiers. The best results have been obtained using Adaptive Random Forest in which Sensitivity reached 96.83% and Precision reached 99.96%.

## 1. Introduction

The rare class problem represents an ongoing challenge for most classification techniques. It results from the imbalanced distribution of target classes in a given data. For binary classification, the data are imbalanced if the quantity of samples belonging to one class is remarkably more than those related to the other. The typical classifiers utilize evaluation measures primarily biased toward the major class. This bias produces misleading quality values in which rare class instances will be classified incorrectly as a major class [1,2]. In many medical applications, the minority is related to the medical condition data instances (for example, the presence of an epileptic seizure or a heart attack); in contrast, most of the data are a normal condition and represents the majority. Misclassifying the disease instance as a normal condition can cause the patient’s health to decline.

Many techniques have tried to solve this problem by increasing the presence of rare class elements (i.e., oversampling) or reducing the presence of the major class elements (i.e., undersampling) [3,4]. Exaggerated oversampling or undersampling can increase false alarms, which means incorrectly classifying the major class instance as a minor class. False alarms reduce the classifier’s reliability; therefore, choosing the appropriate oversampling size represents an additional challenge. However, these techniques focus on handling the data batches where the entire dataset is available as a file before the training of a classifier. In contrast, data arrive as continuous chunks in a data stream, and the presence probability of each class may vary during the training process from one chunk to another.

For classifying chunks of a data stream, a classifier should have a self-adjustment capability to handle the distribution changes and forget old, irrelevant instances [5]. Concept drift refers to these changes, and the ability to track this drift requires the usage of a specific method, such as Adaptive Window ADWIN. It is a sliding window that adapts its size based on the change in the average of samples inside the window. Despite its popularity, ADWIN does not focus primarily on handling an imbalanced data stream. Our earlier work [6] suggested similarity-based window SAW, where the participant instances of an oversampling process are determined based on the existence period and their similarity with the current data. SAW includes a specific period (i.e., the number of prior data chunks) for choosing positive elements of oversampling. This parameter is user-defined, and determining its appropriate value represents a significant limitation of the previous method, making it less applicable. SAW also omits the benefit of selecting positive elements older than the user-specified period. In addition, although SAW selects the nearest elements to the centroid of the current window, it does not consider the amount of distortion in the current set of positive samples, which could impact the data distribution and classification quality. Additionally, SAW uses the actual values of the previous elements (i.e., reinserting old samples into the training process).

In this work, we propose two models of an elastic sliding window based on Cluster Distortion Minimization EWMCD. It is a zero-parameter and self-adjusting window designed to improve the rare class’s presence, enabling an adaptive classifier to classify the minority instances accurately. EWMCD does not require any parameter from the user. The selection process includes evaluating all positive elements from the beginning of the training (i.e., the first data chunk), making it more applicable and leading to more accuracy improvement. The contributions of this work can be characterized as follows:

- It chooses the most valuable previous rare-class instances based on their quality to preserve the coherence of the current data chunk.
- For this task, it utilized the evaluation of Cluster Distortion measurement rather than merely using the mean of values as ADWIN.
- It generates synthetic data points from the real selected candidates inspired by SMOTE (Synthetic Minority Oversampling Technique) to avoid repeating the same old instances; in addition, many of these synthetic points will be closer to the current chuck.
- The implementation utilized the two proposed models to significantly enhance the effectiveness of the adaptive classifiers for the EEG signals. It provides a more independent model for seizure detection with signals generated from wearable sensors of epileptic patients.

## 2. Literature Review

While many methods attempt to address the imbalance in data classes, they are heavily biased towards the rare class by reducing the presence of the major one. This section explains some of the basics of imbalance stream processing, reducing cluster distortion, and synthetic data point generation, also reviewing several related research works.

### 2.1. Classification of Imbalance Data Stream

The binary classification task in supervised Machine Learning (ML) techniques could be represented as $h\left(x\right)\to \hat{y}$, where x refers to the features of an instance. In contrast, $\hat{y}$ is the predicted class for x using the function h. Training these classifiers aims to decrease the difference between the original y and the predicted $\hat{y}$; both of them have only two possible values in the binary classification, such as [0, 1] [7,8]. The probabilities of those two values can be unequal because the samples’ preponderance has a major or negative class value. In contrast, the rare of them have a minor or positive class value [2]. This unbalanced distribution reduces the reliability of the classification model because most classification techniques are majority biased.

On the other hand, the size of data samples can be unlimited in a data stream. Moreover, the distribution of data in time moment T can be different from time T − 1, called the drift concept. The classification model built in T − 1 based on h(xT − 1) could no longer be valid in T. The model should be adapted based on h(xT), and the irrelevant old status should be forgotten [5]. The imbalance class problem becomes more complicated with the data streams because adaptive classifiers focus on the current sample of data, in which the distribution may differ from the overall data distribution. Various techniques have been created to resolve the imbalance in the batch data, and there are attempts to adapt these methods to work with the data stream. Most of these methods depend on undersampling the major class, oversampling the minor class, or merging the two mechanisms [3,4,9].

### 2.2. Synthetic Rare-Class Oversampling

SMOTE method is proposed by [10] to handle imbalanced-class by increasing the presence of rare-class using artificial samples. It begins with selecting one data instance, p1, and one of its nearest neighbors randomly, p2, then generating a synthetic data point in a random position along the line between p1 and p2 [1,11,12]. Due to the effectiveness of this method, many variations have been developed. Borderline-SMOTE is proposed by [13], which used only the wrongly classified instances by the K-NN classifier for SMOTE as border instances [1]. Safe-Level-SMOTE is developed by [14] by filtering the used positive instances according to a safe-level threshold represented by the fraction of the number of p1’s positive k neighbors and the number of positive instances located near these neighbors. The Safe-Level-SMOTE method isolates noise or outlier data points before applying the SMOTE procedure. Another recent method, FW-SMOTE, is represented in [15] that utilized Minkowski distance to specify each positive instance’s neighbor set.

### 2.3. Cluster Distortion Minimization

Many clustering techniques, such as K-means, entail an iterative learning procedure to improve coherence, thereby minimizing the distortion of a cluster [16]. For the data points inside a cluster, the distortion can be calculated using Sum Square Error (SSE) among each cluster’s points and its centroid [17,18]. SSE, in this case, represents the summation of distances SD among data points (p1, p2, …, pn) and the centroid cn as follows [16]:

(1) $$SD=\sum_{1}^{N}{\left[d\left(p_{i},cn\right)\right]}^{2}$$

The elbow method utilizes distortion measurement by SSE to evaluate the effectiveness of the clustering process and choose the appropriate clusters’ number [19,20].

### 2.4. Related Works

Two methods based on undersampling the major class have been presented by [21]. Their strategies included making clusters from the samples of major-class, and the number of those clusters was equivalent to minor-class instances. The centroids of clusters were used as a representative in the first method, while nearest neighbors were utilized in the second method. Their results showed that the second method performed better than five state-of-the-art methods based on 42 datasets. The best results were obtained after combining their approach with a decision tree and single perceptron classifiers. To handle unbalanced class distribution, [22] proposed a hybrid cluster-based instance selection (CBIS) method. It utilized clustering to make subclasses from similar major-class data instances, then separated unrepresentative elements using instance selection. Their implementation evaluated the performance of some ensemble classifiers with CBIS, using two clustering techniques and three instance selection methods; their results showed the quality of CBIS compared with other approaches.

A radial-based method for handling imbalance multi-class has been proposed by [23]. It generated synthetic data instances by utilizing potential functions and considering all classes’ information. Generating the synthetic instances is performed by discovering the areas where the distribution of the mutual class has a small value. The evaluation of their method using 20 datasets showed its usefulness compared to other SMOTE-like algorithms. ADWIN sliding window for extending SMOTE method is applied by [24] to handle the continuous data stream. They applied oversampling procedure for minor-class instances in the current window. Their results showed an improvement in classification performance regarding Recall and F1-Measure.

A framework that utilized dynamic selection for bagging ensemble classifiers is proposed in [25]. The proposed method uses separate sampling with a replacement for the minority and majority classes. Their implementation used 135 artificial data streams artificially with diverse imbalance scales and different levels of label noise and another two real streams. The results of their implementation showed better performance compared with state-of-art methods. Utilization of Hellinger Distance is presented in [26] to improve data stream classification by ensemble pruning. In their method, The base learner in the ensemble is chosen based on the Hellinger Distance, which is determined by calculating TPR and FPR. The poorest model is eliminated if the ensemble size exceeds a pre-defined value; their results showed the usefulness of using Hellinger Distance with some popular classification techniques.

Another method was proposed by [27] in which the data stream was resampled by Poisson distribution in the first step. Another sampling step was used by previously-stored minor-class instances if a high unbalance class state was observed. Their method also dynamically chooses the number of classifiers and utilizes ADWIN for concept drift detection. A transfer learning-based model has been proposed by [28] to handle imbalance classes in real-time data. The model consists of three parts; (1) active sampling that changes the number of samples dynamically, (2) data augmentation to increase data samples and avoid over-fitting, and (3) a DenseNet pre-trained network for transferred learning. Their results showed the effectiveness of the proposed model with both static and real-time data.

## 3. Methodology

The proposed models aim to avoid the bias of the adaptive classifier towards the major class by oversampling the minor class depending on certain previous items. Both models, EWMCD-A and EWMCD-B, consist of two major stages: the first is to select the best positive elements of the prior window that maintain the distribution of the current window. The method has a self-adjusting mechanism to control the candidate set’s appropriate size. This ability automatically manages the increase in the data chunk size due to the rare-class oversampling. The first stage depends on utilizing the criterion of reducing cluster distortion without user intervention. The second stage is creating synthetic elements located at a distance between the current chunk and the elements selected in the first stage of the method. The main difference between the two proposed models is that EWMCD-B eases the conditions in the first stage by choosing all the last chunk’s items (i.e., the previous time moment). EWMCD-B filters the best elements from the oldest windows using the exact mechanism of EWMCD-A. This section will present a detailed description of the EWMCD-A and EWMCD-B steps.

### 3.1. Selecting Elementary MCD Itemset

This stage begins in EWMCD-A with extracting the positive element sets PW(T) and PW(T − 1) from the current chunk W(T) and the previous window W(T − 1). Let PW(T) elements form a virtual cluster VC(T), and CW(T) represents the centroid (i.e., mean of points’ values) of VC(T). As CW(T) represents a reference point for all distance calculations later, finding the centroid and all subsequent steps are performed if the size PW(T − 1) set is more than zero.

The next step is calculating the amount of distortion in the virtual cluster VC(T) by finding SSE between each element inside VC(T) (i.e., PW(T) set) and the centroid point CW(T), then finding MSE for all VC(T) data points. This distortion calculation refers to the coherence of the VC(T) shape, which should be preserved when any previous element from PW(T − 1) is added. On the other hand, the distances among PW(T − 1) elements and the centroid CW(T) are calculated using SSE to evaluate their quality. This evaluation compares the distortion amount in the VC(T) before and after adding each element. Elementary MCD itemset will be formed from PW(T − 1) items that do not cause an increase in MSE after being added to the virtual cluster VC(T). Algorithm 1 summarized this stage of the first model EWMCD-A.
**Algorithm 1** Algorithm of the first stage of EWMCD-A.**Input:** Stream Chunk W(T) and W(T − 1)1:PW(T − 1) = rare-class elements of W(T − 1)2:PW(T) = rare-class elements of W(T)3:Calculate CW(T) of PW(T)4:Calculate SSE(PW(T)) and MSE(PW(T)) between CW(T) and PW(T) items ▹ based on Equation (1)5:Compute SSE(PW(T − 1)) between CW(T) and PW(T − 1) items6:**for each** x ∈ PW(T − 1) **do**7:    Compute MSE of PW(T) in case of including x8:    **if** MSE(PW(T) + x) ≤ MSE(PW(T)) **then**9:        Add x into MCD itemset10:    **end if**11:**end for****Output:** MCD itemset, CW(T)


**Figure 1:**
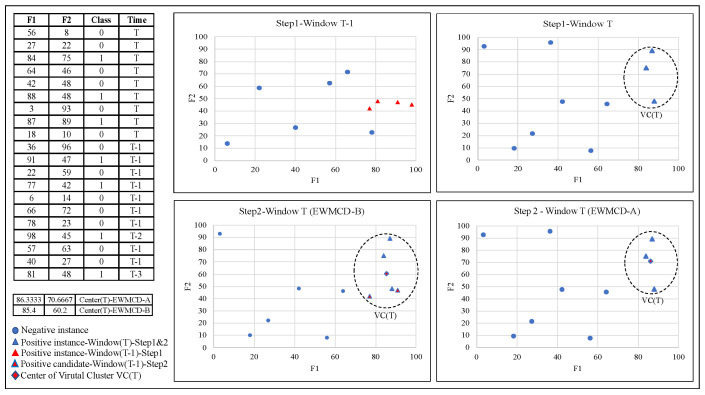
Example of finding the centroid of a Virtual Cluster in EWMCD-A and EWMCD-B.

In the second model, EWMCD-B, VC(T) is formed from the current window positive items PW(T) in addition to some PW(T − 1) items. Due to the oversampling process, the previous window W(T − 1) may contain items from the time moment T − 1 reaching T-z where z refers to chunks’ number before T. EWMCD-B extract only PW(T − 1) items that belong to time moment T − 1 and included them directly to virtual cluster VC(T), then calculates centroid CW(T) and distortion of VC(T). PW(T − 1) remaining items that belong to time moments (T − 2, T − 3, …, T − z) will be evaluated by measuring the distortion using MSE to be part of the elementary MCD itemset. The reason for excluding items of the t − 1 chunk from MCD filtering is that in the t − 1 moment, those items’ centroid was the reference point, and the close items were chosen for it. So, Merging T − 1 items with T without MCD filtering leads to a shift in VC(T) and its centroid CW(T) toward the previous T − 2, … T − z items, thereby increasing the number of oversampling process candidates. Algorithm 2 summarized this stage of the second model EWMCD-B. Moreover, Figure 1 illustrates a numerical example of finding the centroid of a Virtual Cluster in both proposed models.
**Algorithm 2** Algorithm of the first stage of EWMCD-B.**Input:** Stream Chunk W(T) and W(T − 1)1:PW(T − 1) = rare-class elements of W(T − 1) belong to T − 1 time moment2:P$\hat{W}$(T − 1) = rare-class elements of W(T − 1) older than T − 1 time moment3:PW(T) = rare-class elements of W(T)4:**for each** x ∈ P$\hat{W}$(T − 1) **do**5:    Add x into PW(T)6:    Add x into MCD itemset7:**end for**8:Calculate CW(T) of PW(T)9:Calculate SSE(PW(T)) and MSE(PW(T)) between CW(T) and PW(T) items ▹ based on Equation (1)10:Compute SSE(PW(T − 1)) between CW(T) and PW(T − 1) items11:**for each** x ∈ PW(T − 1) **do**12:    Compute MSE of PW(T) in case of including x13:    **if** MSE(PW(T) + x) ≤ MSE(PW(T)) **then**14:        Add x int MCD itemset15:    **end if**16:**end for****Output:** MCD itemset, CW(T)

### 3.2. Generating Synthetic MCD Itemset

This stage utilizes the elementary MCD itemset resulting from the previous stage for both EWMCD-A and EWMCD-B to create Synthetic MCD. It starts by generating a random vector RV for each element within the range [0–1]; the number of values of this vector equals the features’ number of an item in MCD. Then, the distance between the centroid point CW(T) and each element of MCD is calculated.

The next step is generating a synthetic instance (point) $\hat{x}$ that is located on the line between the centroid of the virtual cluster CW(T) and the element x in the MCD itemset. The calculation of features’ values of $\hat{x}$ is performed by adding the product of multiplying the random vector RV(x) by the distance vector DV(x) to the value of the original element x, as in the following equation:

(2) $$\hat{x}=x+\left(DV(x)\times RV(x)\right)$$

**Figure 2:**
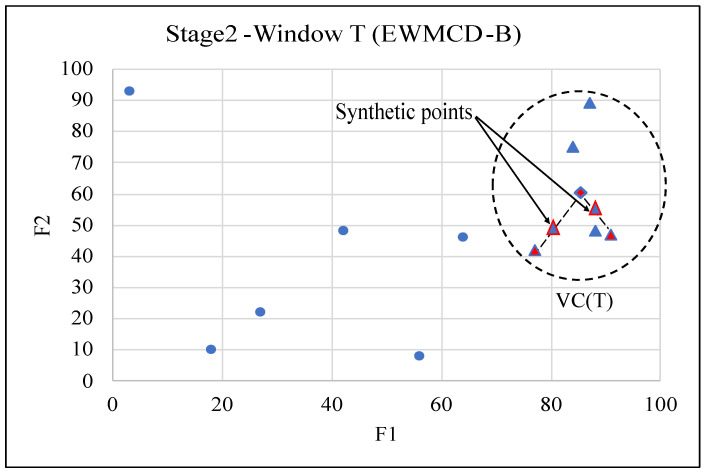
Example of generating two synthetic points in the second stage of EWMCD-B model.

**Figure 3:**
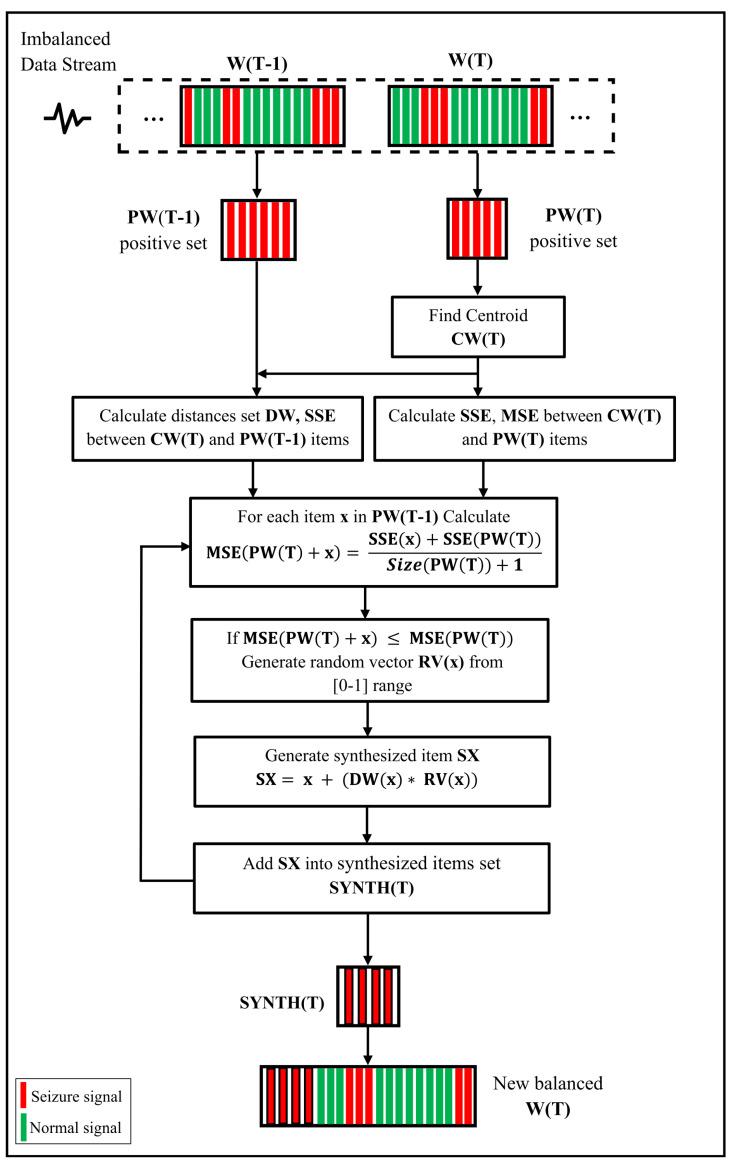
Diagram of EWMCD-A model of self-adjusting window based on minimizing cluster distortion.

The steps of this stage have been summarized in Algorithm 3; in addition, Figure 2 illustrates generating two synthetic points using the EWMCD-B model. All steps of the two stages of the EWMCD-A model are summarized and illustrated in Figure 3.
**Algorithm 3** Algorithm of the second stage of EWMCD-A and EWMCD-B.**Input:** Stream Chunk W(T), MCD itemset, and CW(T)1:**for each** x ∈ MCD **do**2:    Generate random vector RV(x)3:    Calculate Distance Vector DV = C(T)-x4:    Generate synthetic data point $\hat{x}$            ▹ based on Equation (2)5:    Add $\hat{x}$ to synthesized items set SYNTH(T)6:**end for**7:Merge SYNTH(T) with W(T)**Output:** Adapted Window $\hat{W}$(T)


## 4. Implementation and Results

The data extracted from the brain signals of patients with epilepsy represent an essential example of imbalanced medical data. Most of these signals are normal and do not contain an epileptic seizure. In this implementation, a real and big dataset was used as a data stream to evaluate the quality of the proposed method. The evaluation included a number of the popular adaptive classifiers and performance measures that are most used for data stream classification tasks. This section begins with a brief explanation of the used dataset and the framework, then describes the obtained results.

### 4.1. Dataset and Framework Description

EEG signals of the Siena Scalp dataset [29,30] have been used in this implementation. It consists of data from 14 epilepsy patients with a total size of 20.3 GB. Each record contains 35 features obtained from EEG and EKG signals, and the class label is inserted manually in this work based on the seizure time description files. A specific group of files was chosen to prepare 100 chunks of a data stream, each had 1600 instances. PyEDFlib and scikit-multiflow were two major Python libraries used in this experiment. The first one was utilized for feature extraction using FFT, while the second was used for streaming the EEG dataset and adaptive classification tasks.

### 4.2. Experimental Results

The effectiveness of EWMCD-A and EWMCD-B has been evaluated using a performance comparison of five adaptive classifiers with six metrics. The classifiers were Extreme Fast Decision Tree (EFDT), Hoeffding Tree, K Nearest Neighbor (K-NN), OzaBagging, and Adaptive Random Forest (ARF). The metrics were Sensitivity (i.e., True Positive Rate), Specificity (i.e., True Negative Rate), Accuracy, Precision, F-Score, and Matthews Correlation Coefficient (MCC).

**Figure 4:**
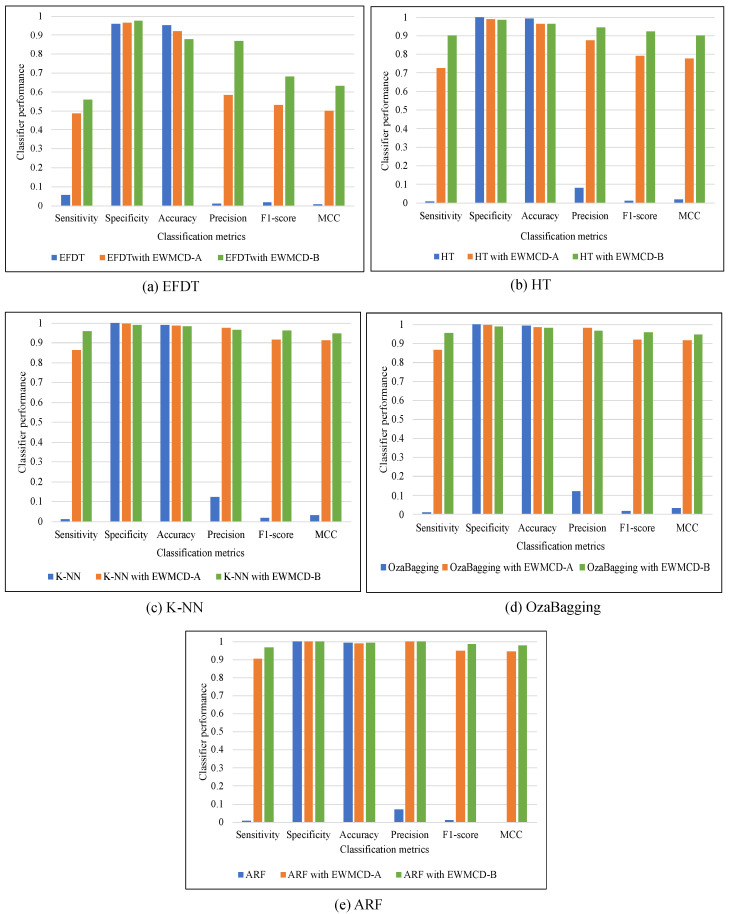
A comparison of five adaptive classifiers using EWMCD-A and EWMCD-B.

The comparison in Table 1 and Table 2 showed that the performance of all classifiers had recognizable improvement using both EWMCD-A and EWMCD-B. Except for the accuracy metric, which tends most to the major class, Precision, F-Score, and MCC confirm this improvement which becomes more apparent in the K-NN classifier. K-NN mainly relies on selecting the closest data points for classification. On the other hand, the ensemble classifiers OzaBagging and ARF benefited more than single model algorithms (i.e., EFDT, Hoeffding Tree), as illustrated in Figure 4. The advantage of ensemble models is resulted from building many base classifiers using different subsets from the given data.

A significant improvement in the performance of ARF can be seen in Table 3. Sensitivity increased from 0.0067 to 0.9016, 0.9682 for EWMCD-A and EWMCD-B, preserving the high value of Specificity in both simultaneously. As a result of this accurate classifying of both classes, the values of Precision enhanced using EWMCD-B from 0.0700 to 0.9996 and F1-score from 0.0122 to 0.9837, representing a notable improvement compared with our previous method SAW. MCC metric can have a more reliable evaluation with an imbalanced dataset [31]; its value increased to 0.9790 using EWMCD-B after it was zero without using the proposed models.

**Figure 5:**
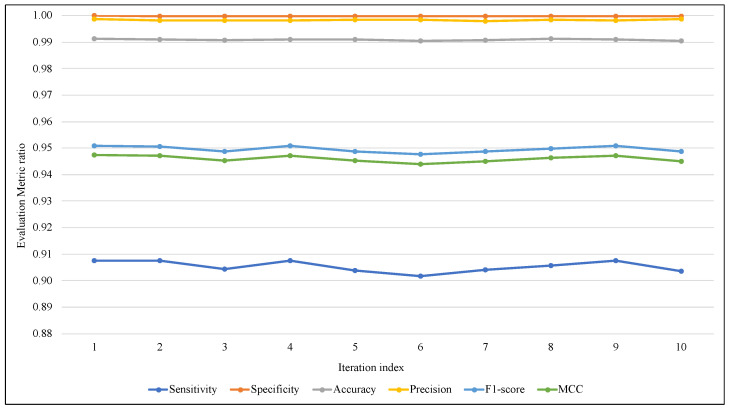
Tracking the effect of randomness on ARF classifier performance using EWMCD-A.

**Figure 6:**
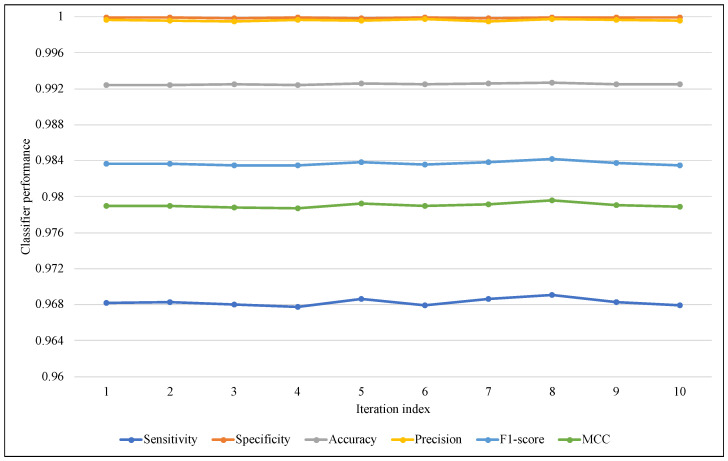
Tracking the effect of randomness on ARF classifier performance using EWMCD-B.

The proposed models use a random function for generating the random vector while creating a synthetic itemset. To avoid the effects of this randomness, in addition to ARF classifier randomness, the test-then-train process has been repeated 10 times, and the average of these iterations was used in comparisons of this section. Figure 5 and Figure 6 illustrate the performance of ARF for all iterations with the six metrics, in Figure 5 of EWMCD-A, although there were slight changes in Sensitivity, the three measures of Precision, F1-score, and MCC remained stable in the 10 trials. In Figure 6 of EWMCD-B, more stability of the ARF classifier can be observed in terms of Sensitivity and the other five metrics as well.

The evaluation also included changing the range of random vector values from [0–1] to [0–0.5] and [0.5–1]. The random vector RV(T) values will be limited between zero and 0.5 in the first range; as a result, the synthetic generated data point will be closer to the centroid CW(T). On the other hand, this point will be toward the MCD item in case of using the second range [0.5–1]. Table 4 showed that ARF had more accurate results using the [0–0.5] range. However, in all the results of other tables and figures, the range [0–1] is used as in the original SMOTE algorithm.

**Figure 7:**
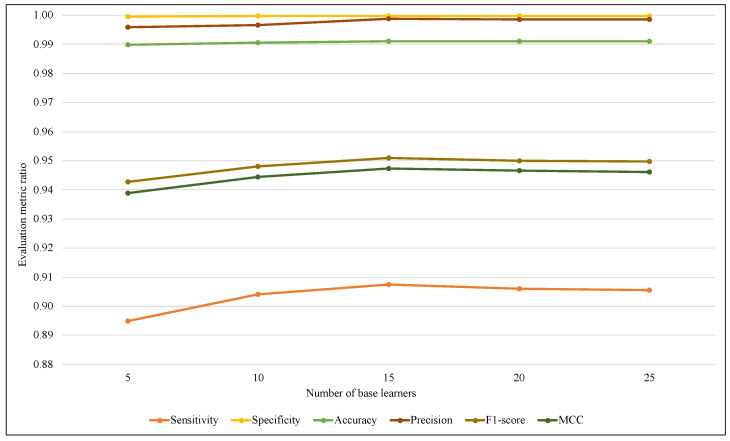
Tracking the effect of an increasing number of base learners on ARF classifier performance using EWMCD-A.

**Figure 8:**
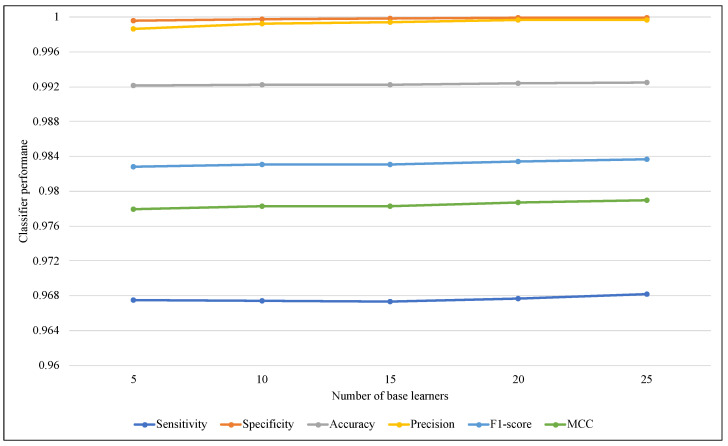
Tracking the effect of an increasing number of base learners on ARF classifier performance using EWMCD-B.

The ensemble size is considered one of the most affected parameters on the ARF classifier that refers to the base learners’ amount. Therefore, another comparison has been performed using different values of ARF ensemble size with the two models. Figure 7 illustrates that using EWMCD-A, ARF effectiveness increased when the number of base learners increased from 5 to 15, then it started to decrease. EWMCD-B had a more stable performance with different values of the ensemble size from 5 to 25, as illustrated in Figure 8, and the best-obtained results were using 25 base learners.

**Figure 9:**
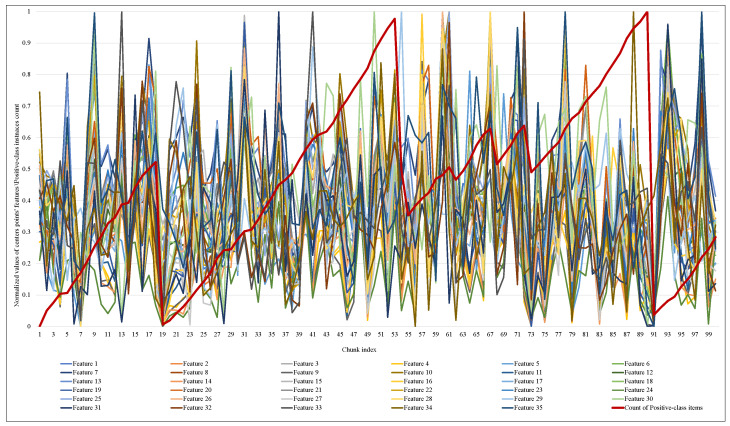
Tracking the response of EWMCD-A window size with changing in values of centroid points in VC.

**Figure 10:**
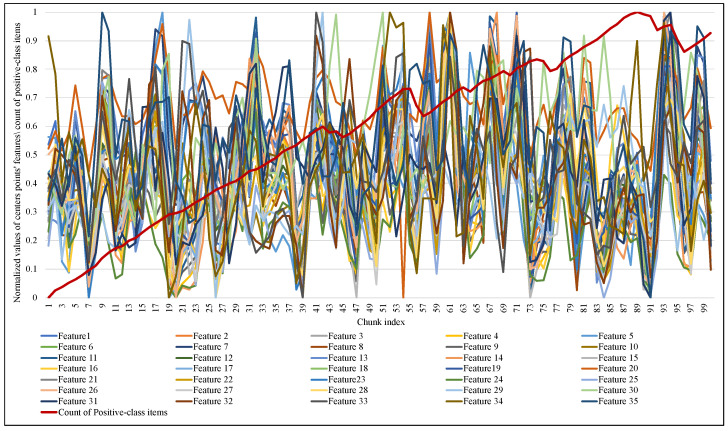
Tracking the response of EWMCD-B window size with changing in values of centroid points in VC.

Another significant observation in this implementation is how and when the size of the proposed elastic window changed and how the performance of the ARF classifier responded to this change. Figure 9 and Figure 10 illustrate the normalized values of the MCD itemset centroid’s features for each data chunk during the training of ARF. Regarding the EWMCD-A model in Figure 9, three major abrupt can be seen in the size of the window in chunk indexes (19, 56, 91). The sudden change in data distribution of the current window reflects on the position of its centroid, thereby reducing the number of elements from the previous window that can be added to the virtual cluster while maintaining the distortion level. In Figure 10, the EWMCD-B model had a more stable window size that grew steadily from the first chunk until chunk 41, where it started to have some drift changes.

**Figure 11:**
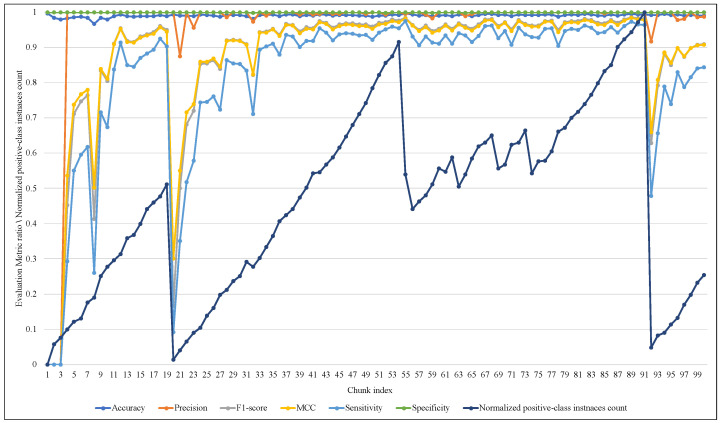
Tracking the response of ARF classifier with changing of EWMCD-A window size.

**Figure 12:**
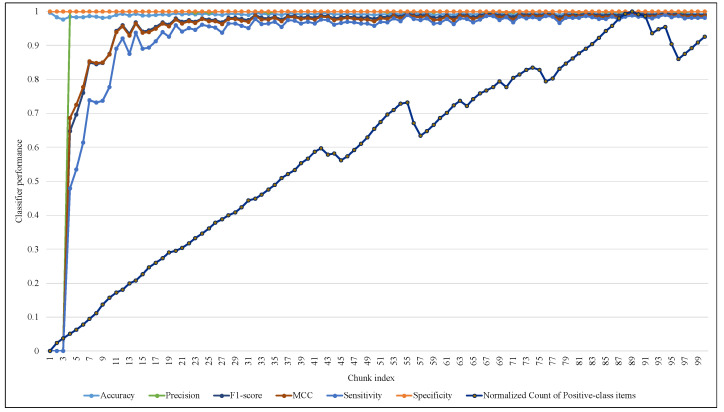
Tracking the response of ARF classifier with changing of EWMCD-B window size.

The rigorous adapting of EWMCD-A and its intensive changes in window size led to a notable response in ARF classifier performance in terms of Sensitivity, F1-score, and MCC while processing the first 40 chunks. After that, ARF had a stable performance, although there were many changes in window size, as Figure 11 showed. On the other hand, EWMCD-B did not suffer similar difficulties, as Figure 12 showed that the classifier had stable performance after chunk 13, up to the end of data stream classification in chunk 100, depending on the values of the six performance measures.

Due to the importance of analyzing and classifying EEG data, the Sienna dataset has been used in many research papers. Table 5 includes a comparison of the performance of the proposed models with four of the most recent studies, noting that the metrics in this table were limited to what has been used in those studies, and their classification models were built as a batch classifier. Regarding accuracy and Specificity, both models EWMCD-A and EWMCD-B had the best results compared with other studies. Furthermore, both models had the best results regarding Sensitivity except for research work [32].

**Figure 13:**
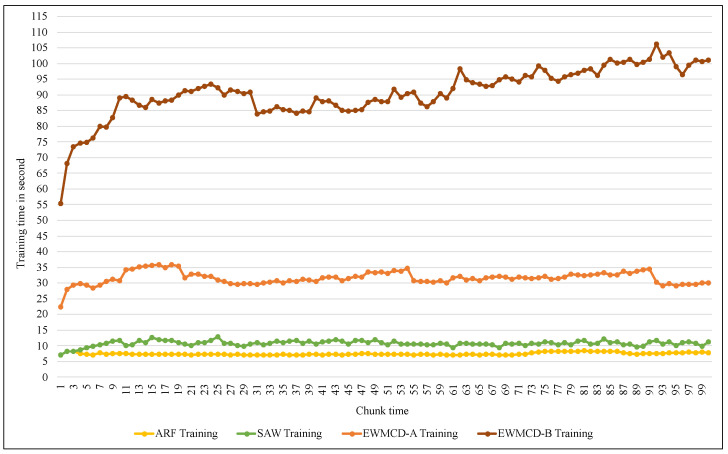
Comparison of required training time of ARF classifier using SAW, EWMCD-A, and EWMCD-B.

The last comparison in the implementation was related to the required computational time for both the training and inference process. Figure 13 illustrates the training time of the ARF classifier using SAW, EWMCD-A, and EWMCD-B models. Despite the increase in the time required by using the EWMCD-A model compared to the original ARF and SAW time, this is due to the number of calculations for measuring distances and generating synthetic data points. The significant increase in training time of the EWMCD-B is related to the accumulative increase in the number of positive-class instances, thereby increasing distances calculations of and modifying the classifier.

**Figure 14:**
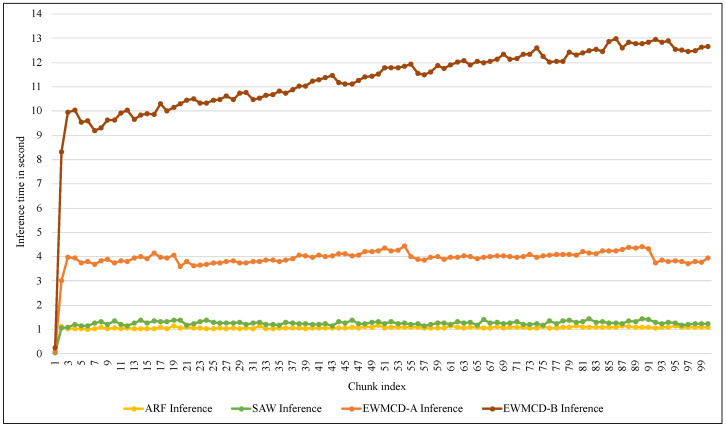
Comparison of required inference time of ARF classifier using SAW, EWMCD-A, and EWMCD-B.

Figure 14 shows similar differences in the time required for the inference process using the proposed models. Although this process does not require calculating distances, using the test-then-train method requires testing each element in the window before training it. Thus, increasing the number of elements means increasing the time needed for inference. Despite this increase, the average time required to infer each instance did not exceed 0.0022 s in EWMCD-A and 0.0054 s in EWMCD-B. This performance provides a quick response to critical medical conditions, such as the early stages of an epileptic seizure, to avoid exacerbating the health condition.

## 5. Discussion

The implementation confirmed the advantage of utilizing the cluster distortion measure to enhance the oversampling of the rare class in the EEG classification and use synthetic data points instead of the original ones. Our findings regarding epileptic seizure detection confirm the usefulness of utilizing the typical oversampling methods, such as SMOTE, to handle imbalance data stream classification with considering its modification to deal with the drift of the data distribution.

While the adaptive classifiers mainly depend on the ADWIN method, this work presented a new method of a special sliding window to handle the imbalanced-class challenge in a data stream. Instead of relying on the average data values inside the window to change its size, the proposed method focuses on the proximity of the previous positive elements to the current stream chunk to preserve the data coherence after enhancing the rare class.

Several improvements and further controls should be considered in the proposed methodology as follows:

- Both models, EWMCD-A and EWMCD-B, are designed to work with imbalanced binary-class tasks, which have only two classes, one of which has the majority. The generalization of its work for multi-class classification needs a modification in selecting MCD items due to the possibility of the existence of more than one rare class and, therefore, the multiplicity of clusters and their centroids. However, using the one-versus-all OVA strategy, the proposed model can be used for the multi-class task without more modification.
- The methodology assumes that all features have numerical values for distance calculations. Applying it to categorical features needs additional preprocessing steps, such as data discretization and binarization.
- The accumulated increase in the number of rare-class samples due to the continuous oversampling may cause a reversal of the classes’ probabilities (i.e., the positive class becomes the major one). This inversion can reduce the efficiency of the adaptive classifier. Therefore, the methodology needs a mechanism to prevent the dominance of the rare class after a period of time from the beginning of the classification process.
- Increasing the number of rare-class samples causes distance computation enlargement, consequently, more classifier adapting time. Models’ efficiency can be enhanced by applying algorithm optimization and parallel and distributed processing techniques.
- The proposed methodology is designed for an instance-labeling environment where the actual value of the class is available immediately or shortly after the inference. The method needs more improvement to handle the delayed-labeling environment.
- Regarding the drift of the data distribution, the extreme sensitivity to this drift by the EWMCD-A model leads to the loss of the accumulated number of rare-class instances and, thus, less stability of the classifier. Despite the good performance of the proposed models, the nature of the EEG data do not contain periodic changes between the positive and negative classes during a specific time, and the effectiveness of these models may decrease with other data types that include a continuous difference between two or more classes, such as human activity data. The EWMCD-B model provided a more stable performance and was less affected by data distribution drift, which may make it more suitable for this type of data.

## 6. Conclusions

The performance of adaptive classifiers could deteriorate because of the imbalanced distribution of classes in a data stream. Although many techniques are available to address this problem in datasets, they cannot be used directly with the data stream due to the unavailability of complete data before the training process. Oversampling the rare class can improve adaptive classifiers’ performance when choosing the appropriate previous items. This work presents a self-adjust window for oversampling the relevant rare-class samples, thereby providing a more reliable classification without undersampling the major class. Minimizing cluster distortion has been utilized as a criterion in the proposed model for filtering the previous positive instances. The implementation included the models’ evaluation based on the Siena EEG dataset. The experimental results showed the ability of both models, EWMCD-A and EWMCD-B, to improve the effectiveness of five adaptive classifiers. ARF classifier obtained the best enhancement in which the F1-score increased from 01.22% to 98.37% using EWMCD-B. Moreover, Precision increased from 7% to 99.96%, and MCC reached 97.9%. Despite the increase in the training time of the two models, the inference time remained within a fast level, as it did not exceed 2.2 milliseconds in EWMCD-A and 5.4 milliseconds in EWMCD-B for each instance. As a future direction, EWMCD can be extended to handle the imbalance of multi-class data stream and delayed labeling environment.

## Figures and Tables

**Table 1:** A comparison of five adaptive classifiers using EWMCD-A.

Technique/Metric	EFDT	Hoeffding Tree	K-NN	OzaBagging	ARF
Sensitivity	0.4876	0.7242	0.8642	0.8677	0.9053
Specificity	0.9646	0.9892	0.9979	0.9981	0.9998
Accuracy	0.9207	0.9639	0.9853	0.9857	0.9909
Precision	0.5824	0.8757	0.9768	0.9798	0.9983
F1-score	0.5308	0.7927	0.9170	0.9203	0.9495
MCC	0.5019	0.7783	0.9109	0.9144	0.9459

**Table 2:** A comparison of five adaptive classifiers using EWMCD-B.

Technique/Metric	EFDT	Hoeffding Tree	K-NN	OzaBagging	ARF
Sensitivity	0.5594	0.9023	0.9566	0.9551	0.9683
Specificity	0.9745	0.9836	0.9890	0.9891	0.9999
Accuracy	0.8776	0.9646	0.9814	0.9812	0.9925
Precision	0.8697	0.9440	0.9638	0.9640	0.9996
F1-score	0.6808	0.9227	0.9602	0.9595	0.9837
MCC	0.6322	0.9001	0.9480	00.9473	0.9790

**Table 3:** Adaptive Random Forest ARF classifier comparison using EWMCD-A and EWMCD-B.

Technique/Metric	ARF	ARF+SAW	ARF + EWMCD-A	ARF + EWMCD-B
Sensitivity	0.0067	0.9313	0.9053	0.9683
Specificity	0.9998	0.9666	0.9998	0.9999
Accuarcy	0.9912	0.9644	0.9909	0.9925
Precision	0.0700	0.6418	0.9983	0.9996
F1-score	0.0122	0.7600	0.9495	0.9837
MCC	0.0000	0.7565	0.9459	0.9790

**Table 4:** ARF classifier Comparison using different ranges for Random Vector RV.

Model/Metric	EWMCD-A			EW-MCD-B		
	[0–0.5]	[0.5–1]	[0–1]	[0–0.5]	[0.5–1]	[0–1]
Sensitivity	0.9260	0.9027	0.9075	0.9702	0.9760	0.9683
Specificity	0.9998	0.9998	0.9999	0.9999	0.9998	0.9999
Accuracy	0.9913	0.9902	0.9911	0.9924	0.9931	0.9925
Precision	0.9987	0.9979	0.9987	0.9998	0.9996	0.9996
F1-score	0.9610	0.9479	0.9509	0.9848	0.9876	0.9837
MCC	0.9570	0.9439	0.9473	0.9799	0.9830	0.9790

**Table 5:** A performance comparison of EWMCD-A and EWMCD-B with four recent articles that used the Sienna dataset.

Articles/ Classification Metric	Accuracy	Sensitivity	Specificity	FPR	FNR
Detti et al. [32] (2018)	96.76	95.71	97.81	-	-
Dissanayakage et al. [33] (2021)	95.88	95.88	96.41	-	-
Sánchez-Hernández et al. [34] (2022)	96	76	-	-	-
Jiang et al. [32] (2023)	95.68	97.51	93.90		
EWMCD-A (proposed method)	99.05	90.16	99.98	00.02	09.84
EWMCD-B (proposed method)	99.25	96.82	99.99	00.01	03.18

## Data Availability

The data presented in this study are openly available in PhysioNet at https://doi.org/10.13026/5d4a-j060.

## Abbreviations

The following abbreviations are used in this manuscript:

EWMCD	Elastic Window based on Minimizing Cluster Distortion
EEG	Electroencephalography
ADWIN	Adaptive Windowing
SMOTE	Synthetic Minority Oversampling Technique
VFDT	Very Fast Decision Tree
EFDT	Extreme Fast Decision Tree
SAW	Similarity-based Adaptive Window
VC	Virtual Cluster
PIA	Positive Instance Age
ARF	Adaptive random forest
TNR	True Negative Rate
TPR	True Positive Rate
FPR	False Positive Rate
FNR	False Negative Rate
